# Autophagic degradation of membrane-bound organelles in plants

**DOI:** 10.1042/BSR20221204

**Published:** 2023-01-16

**Authors:** Jiaojiao Wang, Qian Zhang, Yan Bao, Diane C. Bassham

**Affiliations:** 1 School of Agriculture and Biology, Shanghai Jiao Tong University, Shanghai 200240, China; 2 Department of Genetics, Development and Cell Biology, Iowa State University, Ames, IA 50011, U.S.A.

**Keywords:** Autophagosome, degradation, organelle, receptor, selective autophagy, stress

## Abstract

Eukaryotic cells have evolved membrane-bound organelles, including the endoplasmic reticulum (ER), Golgi, mitochondria, peroxisomes, chloroplasts (in plants and green algae) and lysosomes/vacuoles, for specialized functions. Organelle quality control and their proper interactions are crucial both for normal cell homeostasis and function and for environmental adaption. Dynamic turnover of organelles is tightly controlled, with autophagy playing an essential role. Autophagy is a programmed process for efficient clearing of unwanted or damaged macromolecules or organelles, transporting them to vacuoles for degradation and recycling and thereby enhancing plant environmental plasticity. The specific autophagic engulfment of organelles requires activation of a selective autophagy pathway, recognition of the organelle by a receptor, and selective incorporation of the organelle into autophagosomes. While some of the autophagy machinery and mechanisms for autophagic removal of organelles is conserved across eukaryotes, plants have also developed unique mechanisms and machinery for these pathways. In this review, we discuss recent progress in understanding autophagy regulation in plants, with a focus on autophagic degradation of membrane-bound organelles. We also raise some important outstanding questions to be addressed in the future.

## Introduction

**Figure 1:**
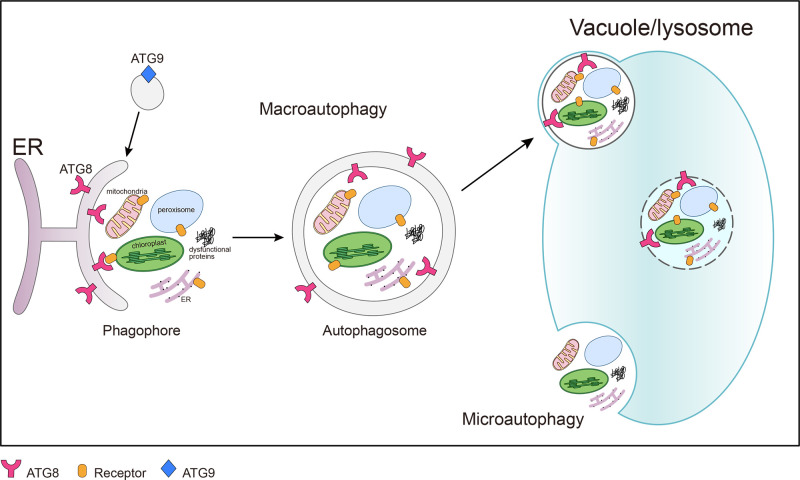
A simplified working model for plant autophagy After the induction of macroautophagy, double membrane structures called phagophores are initiated from the ER with the assistance of ATG9-associated vesicles. The phagophores engulf damaged or excess organelles (e.g. chloroplasts, peroxisomes, mitochondria, ER) or protein aggregates, and transport them to the vacuole for degradation. Alternatively, cytoplasmic cargos may be transported to the vacuole through microautophagy for degradation and recycling.

Autophagy is a fundamental process that is unique to eukaryotes, during which cellular cargoes are targeted for degradation or recycling via the vacuole (yeast and plants) or lysosome (animals) [1,2]. Two types of autophagy are conserved across most eukaryotic species, macroautophagy and microautophagy [3]. During macroautophagy, endoplasmic reticulum (ER)-derived double membrane-bound vesicles called autophagosomes engulf targeted substrates (e.g. dysfunctional proteins or damaged organelles) and deliver them to vacuoles or lysosomes via membrane fusion; while in microautophagy, vacuoles or lysosomes can take up cytosolic substrates directly (Figure 1) [4]. A third type of autophagy has also been described in plants, termed mega-autophagy, during which the vacuole lyses, releasing vacuolar hydrolases into the cytoplasm, resulting in degradation of cellular components and cell death [5]. Activation and progression of autophagy involves many core AuTophaGy (ATG) components and receptors, with multiple distinct steps identified, and has been extensively reviewed [1,2].

Cellular homeostasis requires tight regulation and coordination of various organelles [6]. When homeostasis is disrupted, damaged macromolecules or organelles can be efficiently removed via autophagy [7]. Here, unless otherwise specified, autophagy refers to macroautophagy, as in plants degradation of membrane-bound organelles, the focus of this review, generally occurs via macroautophagy. Selective autophagy of organelles in plants includes ER-phagy, mitophagy, pexophagy and chlorophagy, and requires specific recognition between receptors and their cargo [8]. ATG8 (called LC3 in mammals) is a critical factor that is recruited to and tethered on the membrane of autophagosomes via covalent conjugation to the membrane lipid phosphatidylethanolamine. Binding of cargo receptors to ATG8 then recruits the receptor and cargo into the autophagosome for transport and degradation. Multiple ATG8 isoforms (9 copies in Arabidopsis) are present in plants, potentially allowing distinct regulatory mechanisms for autophagy during growth and stress responses [9]. ATG8 proteins interact with receptor proteins through specific motifs, and an ATG8-interacting motif (AIM) is present in most ATG8-interacting proteins involved in organellar autophagy [10,11] (Table 1).

**Table 1:** Receptors for autophagic degradation of membrane-bound organelles

Autophagy type	Receptors	Stimuli	References
ER-Phagy	ATI1	Carbon starvation, viral infection	[36,38]
	ATI2	Carbon starvation, viral infection	[36,38]
	RTN1	ER stress	[30]
	RTN2	ER stress	[30]
	Sec62	ER stress	[26]
	C53	Stalled ribosomes, ER stress	[34]
	RHD3	ER stress	[24]
Mitophagy	FMT	Uncoupler DNP	[53]
	TraB1	Uncoupler DNP	[54]
Pexophagy	NBR1	Cadmium stress	[66,68]
	PEX10	na	[69,70]
	ABCD1/PXA1	ROS	[64]
	ARP2/3	NAA and 3-MA	[74]
Chlorophagy	ATI1	Carbon starvation, heat stress	[97,98]

na, not applicable.

## ER-phagy

### ER-phagy and ER stress

The ER is a dynamic and continuous membrane system in eukaryotic cells. It is a highly expanded structure, with multiple morphologies, including the nuclear envelope, rough ER (RER) sheets with ribosomes, and smooth ER (SER) tubules connected by three-way junctions [12]. These different structures facilitate distinct ER functions, including RER-mediated protein synthesis, folding and vesicle transport, SER-mediated lipid production, and communication with other organelles. Meanwhile, the ER is continuously undergoing highly dynamic morphological remodeling in response to different environmental stimuli, allowing stress adaptation and recovery [13]. When the processing and protein folding capacity of the ER is overloaded, it will cause unfolded protein accumulation, a situation termed ER stress [14]. Organisms have evolved strategies to deal with ER stress, including ER-associated degradation (ERAD), the unfolded protein response (UPR), and ER-phagy, an important pathway that degrades ER fragments or ER-associated components. ER-phagy is a selective process that involves the autophagic machinery and corresponding receptors to accomplish the vacuolar degradation of ER [15].

In plants, ER stress-mediated ER-phagy is triggered by the accumulation of misfolded proteins in the ER [16]. ER fragments were observed in autophagic bodies upon treatment with the ER stress agent tunicamycin (Tm), and the ER stress sensor IRE1b (inositol-requiring enzyme 1b) is required for this process [17]. IRE1b has two major activities, non-conventional splicing of the mRNA of the transcription factor bZIP60 (basic region/leucine zipper motif 60) that in turn activates ER stress-response gene transcription, and regulated IRE1-dependent mRNA decay (RIDD), a general mRNA degradation pathway that reduces production of ER proteins and therefore relieves ER stress. The ribonuclease activity of IRE1b was found to be critical for IRE1b-mediated autophagy during ER stress [18,19], and this was due to RIDD activity rather than *bZIP60* splicing, demonstrating RIDD-dependent and bZIP60-independent regulation of ER-phagy [19].

Other regulators of autophagy during ER stress have been identified. SnRK1 (SNF1-related protein kinase 1) is a protein kinase that senses the energy status of the cell [20] and is required for activation of autophagy under many stress conditions, including ER stress [21]. How energy status and ER stress are linked, how autophagy activation is triggered by SnRK1, and how IRE1b and SnRK1 activities are coordinated is unknown. Sulfide has also been shown to negatively regulate ER-phagy, via persulfidation of the autophagy core factor ATG18a [22]. While ATG18a is required for bulk autophagy under various stress conditions, its regulation by persulfidation seems to be restricted to ER stress conditions. Persulfidation increases binding of ATG18a to phosphatidylinositol 3-phosphate, which then controls the number and size of autophagosomes produced upon ER stress. Other Arabidopsis ER-associated proteins are potentially involved in ER-phagy, such as NAP1 (Nck-associated protein 1). NAP1 was found to be involved in autophagosome biogenesis by affecting actin nucleation [23]; a potential role for NAP1 in ER-phagy regulation is an interesting topic for future investigation.

### ER-phagy receptors during ER stress

**Figure 2:**
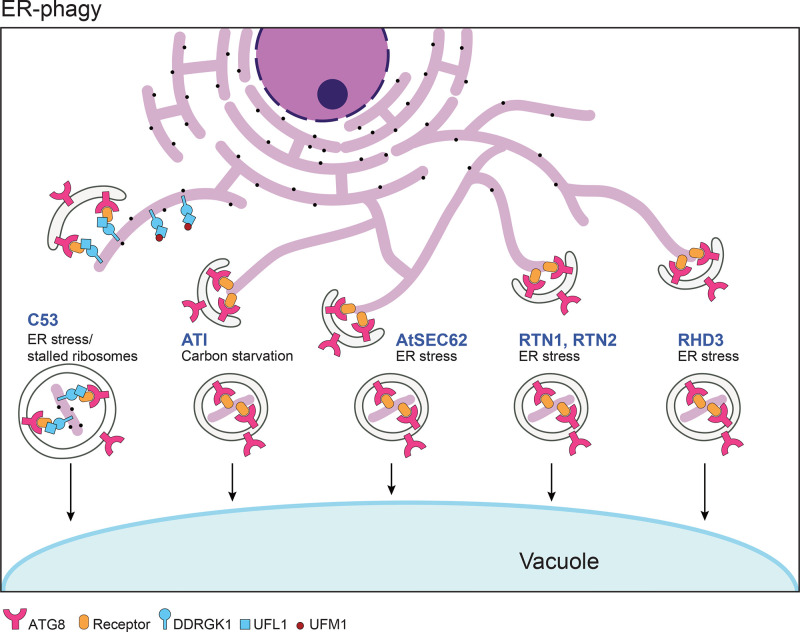
A working model for ER-phagy in plants Multiple routes govern the degradation of ER fragments or its associated components during ER-phagy. As a response to certain stressful stimuli (e.g. carbon starvation or ER stress), specific ER-phagy receptors including C53, ATI, Sec62, RTN, and RHD3, are employed for selective degradation of ER-associated targets.

ER-phagy relies on specific receptor–adaptor interactions to facilitate engulfment of ER fragments by autophagosomes or direct delivery to the vacuole. To date, many ER-phagy receptors were identified and characterized in eukaryotes, including FAM134, Sec62, RTN3, CCPG1, ATL3, TEX264, CALCOCO1 and C53 in mammals [13]; Atg39, Atg40, and Epr1 in yeast [13]; and ATI1, ATI2, ATI3, RTN1, RTN2, AtSEC62, C53 and RHD3 in plants [13,24]. Different receptors can perceive distinct signals to control the degradation of ER fragments (Figure 2), indicating their functional diversification in ER-phagy.

SEC62 is a component of the translocon complex, and was initially identified in mammals as an ER-phagy receptor during stress recovery [25]. Arabidopsis AtSEC62 has translocon domains but only shares 12% and 15% protein sequence similarity with its counterparts in yeast and animals, respectively, and has a unique membrane topology, suggesting potential functional differences. AtSEC62 is ER membrane-associated and interacts with ATG8 through its AIM motif during ER stress triggered by Tm or dithiothreitol (DTT) [26], Interestingly, ring-like structures marked by YFP-AtSEC62 and the autophagosome marker mCherry-ATG8e were observed upon ER stress induction. *atsec62* null alleles were sensitive to Tm, whereas overexpression of AtSEC62 enhances stress tolerance [26], raising the hypothesis that AtSEC62 can act as a receptor in ER stress-regulated autophagy.

Reticulons (RTNs) are ER-localized transmembrane proteins with a highly conserved reticulon homology domain [27]. In mammals, two reticulon domain-containing proteins, FAM134B and RTN3 were characterized as ER-phagy receptors in mediating ER turnover [28,29]. In plants, maize RTN1 and RTN2 proteins were reported to be ER-phagy receptors, containing four AIM motifs, and the interactions between RTN and ATG8 were enhanced upon ER stress treatment [30]. In endosperm cells of maize *rtn2* mutants, autophagy induction and up-regulation of ER stress-responsive chaperones were detected, suggesting that ER homeostasis was disrupted, and therefore indicating a crucial role of maize RTN1- and RTN2-controlled ER-phagy in ER homeostasis and stress [30].

Arabidopsis ROOT HAIR DEFECTIVE (RHD) 3 is an atlastin GTPase previously reported to be involved in root development [31], and more recently identified as an ER-phagy receptor [24]. The orthologs of RHD3 in mammals, atlastin 2 (ATL2) and 3 (ATL3), were reported to play an important role in ER-phagy [32,33]. ATL2 is required for FAM134B-mediated ER-phagy [32] and ATL3 functions as a receptor for ER-phagy, interacting with the ATG8-related protein GABARAP to promote tubular ER degradation upon starvation [33]. Two distinct AIM sites were identified on RHD3, but interestingly, only AIM2 is involved in the interaction with ATG8, and ER stress treatments enhance the interaction between RHD3 and ATG8. Sun et al. [24] further showed that an *rhd3* mutant is sensitive to ER stress and deficient in ER-phagy.

C53 is a unique ER-phagy receptor conserved in both plants and animals. First, it is a cytosolic protein, unlike most other ER-phagy receptors, which are ER membrane-localized. Second, it interacts with ATG8 via a shuffled ATG8 interacting motif (sAIM), rather than a conventional AIM site. Third, it forms a tripartite receptor complex with the ER-associated ufmylation ligase UFL1 and its membrane adaptor DDRGK1 to sense the proteotoxic level in the ER lumen; the complex is activated by stalled ribosomes at the ER surface [34]. This discovery suggests that ER-phagy receptors can have diverse cellular localizations, that the motif for interacting with ATG8 is not necessarily conserved, and that helper proteins can be recruited to form complexes to mediate ER-phagy.

### ER-phagy receptors during other types of stress

Beyond ER stress [35], dark-induced starvation [36], phosphate starvation [37] and viral infection [38] were also reported to induce ER-phagy in plants. In many cases, the specific receptor that recognizes the ER is unknown.

ATI1 (ATG8-interacting 1) and ATI2 are plant-specific ATG8-binding transmembrane proteins that were found to be involved in ER-phagy [36,38]. ATI proteins contain two putative AIM sites [39], located in the long intrinsically disordered regions (IDRs) at the N-terminus [40]. During dark-induced carbon starvation, ER-localized ATI proteins associate with ER-derived bodies and sequester these bodies for autophagic degradation in the vacuole. In addition, ATI proteins can interact with MSBP1 (membrane steroid-binding protein 1) and facilitate its degradation through ER-phagy during carbon starvation [36]. The ATI proteins also interact with AGO1 (argonaute 1) protein on the ER, leading to its vacuolar degradation, playing a critical role in plant–virus interactions [41]. ATI3 is a dicot-specific protein that was initially isolated as an ATG8-interacting protein from a yeast-two-hybrid screen [42,43]. ATI3 interacts with ER-localized UBAC2 (Ubiquitin-associated protein 2) protein, leading to its vacuolar degradation in an autophagy-dependent manner.

## Mitophagy

Mitochondria are double membrane-bound organelles within eukaryotic cells that serve as the powerhouse by generating adenosine triphosphate (ATP). Many additional biochemical activities are carried out in mitochondria, including *de novo* fatty acid synthesis, amino acid biosynthesis, and iron–sulfur biosynthesis [44]. Mitochondria are also major sources of reactive oxygen species (ROS) that can result in oxidative damage, and this ROS production increases when mitochondria are damaged. Therefore, maintaining a healthy mitochondrial population is important for plant cells, ensuring energy supply and multiple biochemical activities, and preventing excess ROS production [45]. To maintain cell homeostasis, autophagic clearance of damaged or superfluous mitochondria (mitophagy) is critical.

**Figure 3:**
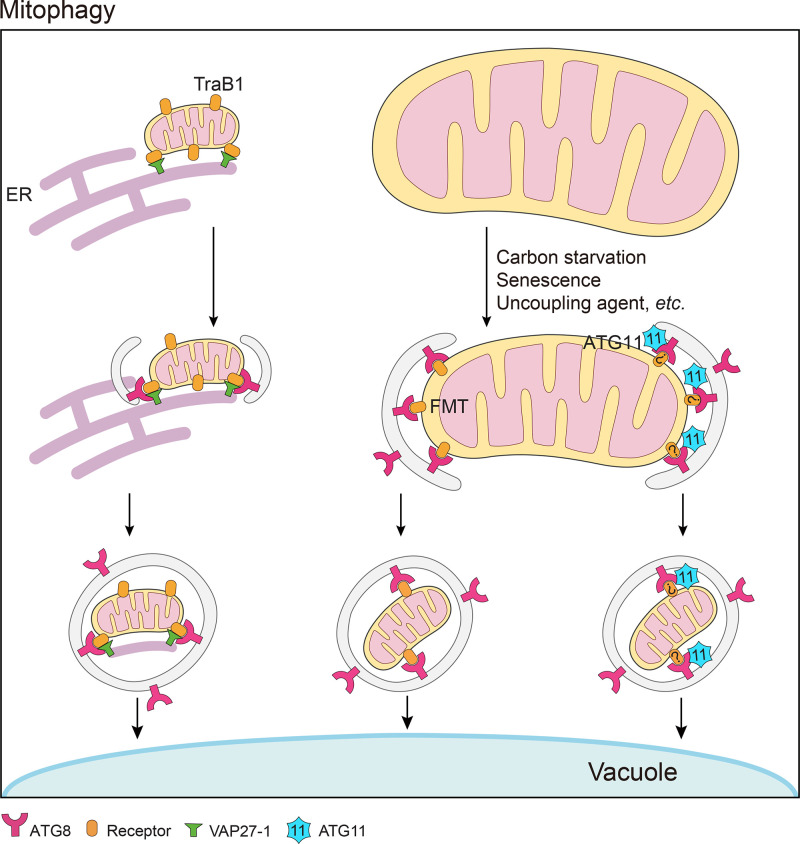
A working model for mitophagy in plants Selective degradation of mitochondria can be carried out through two main routes in plants. Targeted mitochondria can be first tethered to the ER via interaction between TraB1 and VAP27-1 and then recognized by the autophagy adaptor ATG8; or they can be directly recognized by ATG8 via the specific receptor Friendly (FMT), or via unknown receptors and ATG11.

Based on the mechanism of recognition of mitochondria for degradation, mitophagy can be classified into three types: (1) ubiquitin-dependent, (2) receptor-dependent and (3) lipid-dependent [45]. Mitophagy is best described in mammals, where ubiquitylation (e.g. via the E3 ubiquitin ligase PARKIN and PTEN-induced kinase 1, PINK1), receptors [such as FUN14 domain-containing protein 1 (FUNDC1), BCL2 Interacting Protein 1 (BNIP1) and NIX] and lipids (cardiolipin and ceramide) can be the selective signals to mark damaged mitochondria and recruit LC3 to allow autophagic degradation [45]. In yeast, the mitophagy receptor ATG32 is activated by casein kinase 2 via phosphorylation, binds ATG11 and then interacts with ATG8 [46,47]. Compared with the studies in yeast and animals, mechanisms of selective mitophagy in plants are still largely unknown (Figure 3). In addition, very few of the major participants of mitophagy in animals and yeast mentioned above have clear orthologs in plants.

### Regulation of mitophagy in plants

A variety of environmental stimuli, including senescence, carbon or nitrogen starvation, or UV-B stress, can trigger mitophagy in plants. For instance, the number of mitochondria and amount of mitochondrial protein decreased significantly in senescent leaves of wild-type (WT) Arabidopsis plants but were stabilized in the autophagy deficient mutants *atg7* and *atg11*. When leaves were pretreated with the vacuolar H^+^-ATPase inhibitor concanamycin A (ConcA), mitophagic bodies marked by Mito-YFP and mCherry-ATG8a became visible in individually darkened leaves of WT Arabidopsis plants, but were absent from the leaves of *atg7* or *atg11* mutants [48]. ATG11 is an autophagy adaptor that can interact with ATG8 through its AIM motif and, together with ATG7, participate in senescence-induced mitophagy in Arabidopsis [48]. In another study, autophagic bodies containing mitochondria were found in roots under nitrogen starvation upon ConcA treatment, but were not seen in the autophagy deficient mutant *atg4a atg4b* [49]. A high dosage UV-B stress can cause mitochondria to be inactivated and fragmented, and mitophagy was reported to play an important role in autophagic clearance of damaged mitochondria through vacuolar degradation [50,51].

Mitophagy can also be triggered by a range of mitochondrial inhibitors, such as doxycycline (Dox, inhibits translation on mitochondrial ribosomes), MitoBlockCK-6 (MB, inhibits mitochondrial protein import), and carbonyl cyanide-p-trifluoromethoxyphenylhydrazone (FCCP) and 2,4-dinitrophenol (DNP), uncouplers which depolarize mitochondria [52,53]. Of note, adding those inhibitors to the growth medium leads to a more pronounced mitophagy flux than spraying on plants. In addition, as an uncoupler, FCCP was more potent than DNP, depolarizing almost all mitochondria at a lower concentration, making it very challenging to monitor mitophagy dynamics. For this reason, DNP is the more widely used uncoupler because its slower action facilitates the observation of mitophagy flux via cell biological and biochemical assays [53,54].

Kacprzak et al. [52] established a new system to monitor mitophagy levels in plants by generating a stable Arabidopsis transgenic line expressing GFP fused with the mitochondrial matrix-localized isocitrate dehydrogenase 1 (IDH1) or mitochondrial outer membrane localized Translocase of Outer Membrane 20 (TOM20). With these new reporter lines, they found that dark-induced carbon starvation, natural senescence, and specific mitochondrial stresses (long-term exposure to uncoupling agents or inhibitors of mitochondrial protein import/translation) are key triggers of mitophagy in plants, while nitrogen starvation, hydrogen peroxide, heat, UV-B and hypoxia did not appear to trigger substantial mitophagy [52]. These findings provide new tools to detect mitophagy in plants and demonstrate effective inducing conditions or treatments.

### Recognition of mitochondria for degradation

Ma et al. [53] recently reported that Friendly (FMT), a member of the clustered mitochondria protein family, translocates to damaged mitochondria to mediate uncoupler-induced mitophagy. Upon treatment with the uncoupler DNP, *fmt* mutants have more depolarized mitochondria and fewer mitophagosomes, indicating that FMT is critical for mitophagy [53]. Defects were also observed in mitophagy during cotyledon greening, identifying a physiological role for FMT in development. However, how Friendly promotes autophagosome formation with its potential binding partners require additional research.

When mitophagy is activated in response to environmental or physiological cues, for example during pollen tube growth [55], the mechanism for distinguishing damaged mitochondria from the functional population is crucial for selective autophagic degradation. TraB1, an uncharacterized mitochondrial outer-membrane protein, was identified as a novel ATG8-interacting component in mitophagy. Interestingly, the ER-localized protein VAP27-1 (vesicle-associated protein 27-1), can directly interact with TraB1 and regulate its ER-mitochondrial tethering and turnover through mitophagy [54], indicating that distinct mechanisms exist for control of mitophagy in plants.

## Pexophagy

**Figure 4:**
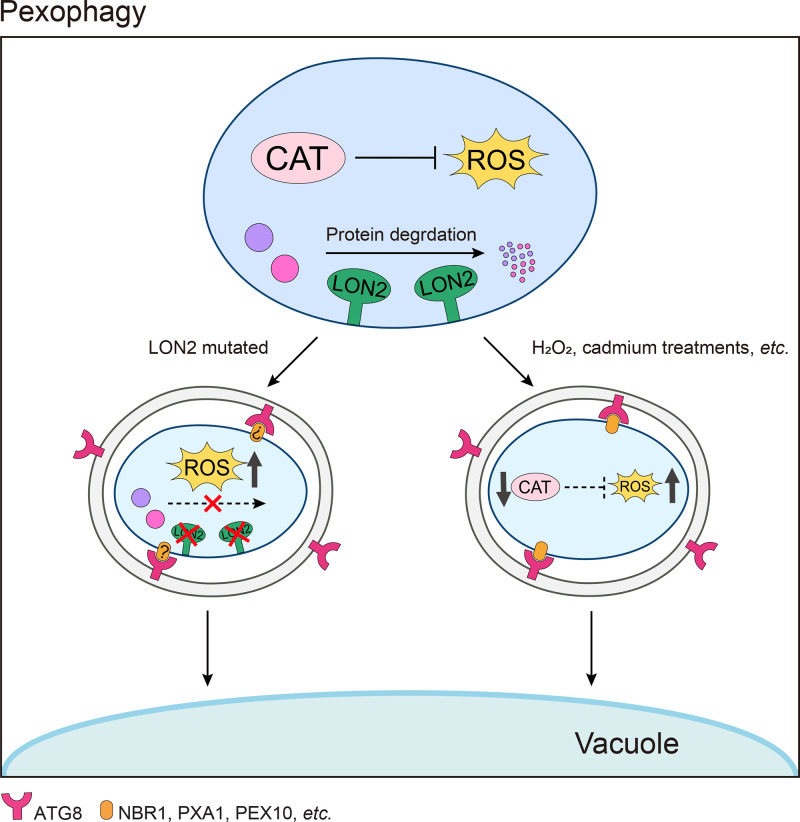
A working model for pexophagy in plants Imbalance of ROS homeostasis (cadmium or other stress treatments) or a genetic defect (LON2 mutation) in peroxisomes causes pexophagy-mediated vacuolar degradation via various specific receptors including NBR1, PXA1, PEX10 or DSK2.

Peroxisomes are small, single membrane organelles with diameters of approximately 0.1–1 μm. Despite their simple structure and small size, peroxisomes contain over 200 proteins, involved in diverse metabolic functions [56]. In seeds, glyoxysomes, a specialized form of peroxisomes, function in β-oxidation and the glyoxylate cycle, converting lipids into sucrose to support post-germination growth of seedlings. In leaves, peroxisomes are involved in photorespiration, ROS catabolism, and production of hormones, including auxin, jasmonic acid and salicylic acid, which are essential phytohormones for plant growth and stress responses. Autophagic degradation of peroxisomes, termed pexophagy (Figure 4), is required for the conversion of the population of peroxisomes from seed glyoxysomes to leaf peroxisomes, and for their quality control to remove damaged peroxisomes [57].

### Pexophagy in development and stress responses

Glyoxysomes are directly transformed into leaf peroxisomes during the greening of etiolated cotyledons for seedling peroxisome remodeling [58], along with the degradation of obsolete glyoxysomal proteins such as isocitrate lyase (ICL) and malate synthase (MLS), two marker enzymes of the glyoxylate cycle [59]. In the autophagy-deficient mutants *atg5* and *atg7*, more peroxisomes and endogenous glyoxysomal proteins (such as ICL and MLS) accumulate in the hypocotyls of developing seedlings. Furthermore, when the seedlings were treated with ConcA, peroxisomes were found in the vacuole of WT hypocotyls but not in that of the *atg7* mutant, indicating that pexophagy participates in the degradation of glyoxysomal proteins [60]. During this functional transition of peroxisomes, unnecessary proteins are degraded by both LON2 (LON protease 2)- and autophagy-dependent pathways. LON2 belongs to the AAA+ (ATPases associated with various cellular activities) superfamily, and can act as both an ATP-dependent protease and a chaperone. *lon2* mutants have defects in peroxisomal number and metabolism and in protein import, and these defects are suppressed by *atg* mutants, indicating that pexophagy and LON2 cooperate in peroxisome quality control [61,62].

Under normal growth conditions, plants maintain a basal level of pexophagy, as autophagy-deficient mutants have increased numbers of peroxisomes compared to WT plants [57,60]. Treatment of tobacco BY2 cells with the autophagy inhibitor 3-methyladenine (3-MA) led to accumulation of peroxisomes and peroxisomal proteins [63]. Pexophagy is also involved in plant responses to various stressful conditions. In BY2 cells, the number of peroxisomes dropped substantially during sucrose starvation, and 3-MA delayed peroxisome degradation, indicating that carbon starvation effectively triggers autophagic degradation of peroxisomes [63]. Under high glucose treatment (3%), the autophagy-deficient mutants *atg5* and *atg7* accumulate more peroxisomes in root cells than do WT plants, indicating that high glucose-promoted peroxisome degradation in roots requires a functional autophagy pathway [64].

Peroxisomes generate ROS, which need to be removed by antioxidant enzymes such as catalase. When ROS accumulation in peroxisomes causes oxidative damage of peroxisomal proteins or other peroxisomal components, the resulting dysfunctional peroxisomes need to be removed. Although the signals that trigger plant pexophagy have not yet been well characterized, oxidative changes seem to be a key factor. Using unusual positioning of peroxisomes as a criterion, Shibata et al [65] identified several peroxisome unusual positioning (*peup*) Arabidopsis mutants, which were found to be mutated in *ATG2*, *ATG18a* and *ATG7* genes. In *peup*/*atg* mutants, oxidized peroxisomes accumulated in large aggregates and contained inactive catalase; these aggregates were also found in a catalase mutant. Damaged and aggregated peroxisomes are therefore degraded by autophagy as a quality control mechanism [65]. Even under normal growth conditions, peroxisomes in leaf cells of autophagy mutants contained increased levels of catalase in an insoluble and inactive aggregate form, and these accumulated abnormal peroxisomes were selectively recognized and delivered to vacuoles for degradation upon restoration of autophagy function [57]. Similarly, exposure of Arabidopsis plants to cadmium induces oxidative stress, and oxidation of peroxisomal proteins such as catalase is likely a trigger for pexophagy [66].

### Identification of pexophagy machinery

The mechanistic understanding of pexophagy has been increasing over the last few years. In yeast, the major players for recognition of peroxisomes for degradation are Atg36 and Atg30, while mammals use p62/SQSTM1 or NBR1 as pexophagy receptors [67]. Plants have no clear counterparts of Atg36 or Atg30 but may use the conserved component NBR1 as a peroxisome receptor. In cadmium-induced pexophagy in Arabidopsis, NBR1 co-localizes with ATG8 and catalase, suggesting that NBR1 may function as a pexophagy receptor [66]. However, Young et al. [68] showed that NBR1 is not required for pexophagy in the *lon2* mutant, and overexpression of NBR1 is not sufficient to trigger pexophagy, suggesting that an NBR1-independent mechanism for pexophagy also exists in Arabidopsis. Through bioinformatics approaches, Xie et al. [69] identified nine peroxisomal PEX proteins in Arabidopsis that contain high fidelity AIMs (hfAIMs), among which AtPEX6 and AtPEX10 interact with ATG8 *in vivo* as validated by bimolecular fluorescence complementation (BiFC). Moreover, mutations occurring within or near hfAIMs in PEX6 and PEX10 cause defects in the growth and development of various organisms, indicating that the conserved hfAIMs are important for their functions [69]. In addition, an independent yeast two-hybrid screen also identified PEX10 as an ATG8-interacting protein [70], suggesting that PEX10 is a promising candidate for a pexophagy receptor.

ABCD1/PXA1 (ATP-binding cassette D1; Formerly PXA1/peroxisomal ABC transporter 1) is a peroxisomal transmembrane protein, and plays multiple roles in plant lipid metabolism and signaling, including the transport of indole-3-butyric acid (IBA) for subsequent conversion via β-oxidation into the active auxin indole-3-acetic acid (IAA) [56]. The Walker B motif of ABCD1/PXA1 physically interacts with ATG8e *in vitro* and *in vivo*, as verified by yeast two-hybrid and coimmunoprecipitation assays [64]. In addition, overexpression of ABCD1 partially rescues the glucose-associated phenotypes of the *atg* mutants. Therefore, ABCD1/PXA1 is another possible receptor for pexophagy. The ubiquitin-binding protein DSK2 (dominant suppressor of KAR2) was proposed as another pexophagy receptor/adaptor candidate in plants [71–73]. DSK2 functions in autophagy by interacting with ATG8 through its AIM sites [72]. DSK2 also interacts with the RING (really interesting new gene) finger domain of two peroxisomal membrane proteins, PEX2 and PEX12 [71]. However, DSK2 is not a peroxisome-associated protein, and there is no clear evidence that PEX2 or PEX12 recruit DSK2 to peroxisomes. Thus, the role of DSK2 in plant pexophagy needs to be verified. Finally, ARP2/3 (Actin Related Protein 2/3 complex) is a heteroheptameric protein that participates in actin reorganization at the plasma membrane (PM) and at PM-ER contact sites. Martinek et al. [74] recently found that ARP2/3 complex-containing dots associate exclusively with peroxisomes in plant cells, and co-localize with the autophagosome marker ATG8f under autophagy-inducing conditions. Moreover, ARP2/3 subunits co-immunoprecipitate with ATG8f, and mutants lacking functional ARP2/3 complex have more peroxisomes than do WT plants. ARP2/3 may therefore function as a receptor or adaptor in pexophagy [74].

## Chlorophagy

Chloroplasts are specialized plastids found in plants and algae in which photosynthesis converts light and CO_2_ into chemical energy and carbohydrates to support their photoautotrophic lifecycle. Mature chloroplasts contain two envelope membranes (outer and inner), a soluble stroma and a thylakoid membrane system. Starch granules are often present in the stroma as a product of photosynthesis, and chloroplasts also contain numerous proteins and metabolites [75].

Turnover of chloroplasts must be tightly controlled to maintain photosynthetic function and alleviate cell damage. Chloroplasts are degraded during leaf senescence to remobilize their contents, and also upon environmental stress, as removing damaged chloroplasts is critical in maintaining cell viability [76]. Photo-oxidative damage of chloroplasts is frequently encountered, caused by photosynthesis-related superoxide (O^2−^), hydrogen peroxide (H_2_O_2_) and singlet oxygen (^1^O_2_) or ROS produced upon exposure to UV-B or high light (HL) [76]. Chloroplasts are highly sensitive to different stresses, including carbon starvation, salt stress and the combination of abnormal light with low or high temperature. Senescence or stress often causes changes to chloroplast morphology along with the decrease in photosynthetic efficiency. Chloroplasts in senescing leaves often have more and bigger plastoglobules (lipoprotein particles), collapsed thylakoid membranes and disrupted envelope [77]. Upon strong UV-B exposure for a short period, chloroplasts become smaller but have larger plastoglobules, and the number of chloroplasts decreases significantly [78]. The structure of the thylakoid system in particular is dynamic in response to different light intensities [75]. These features indicate that quality control of chloroplasts is essential to maintain normal plant growth and development.

### Pathways for chloroplast turnover

**Figure 5:**
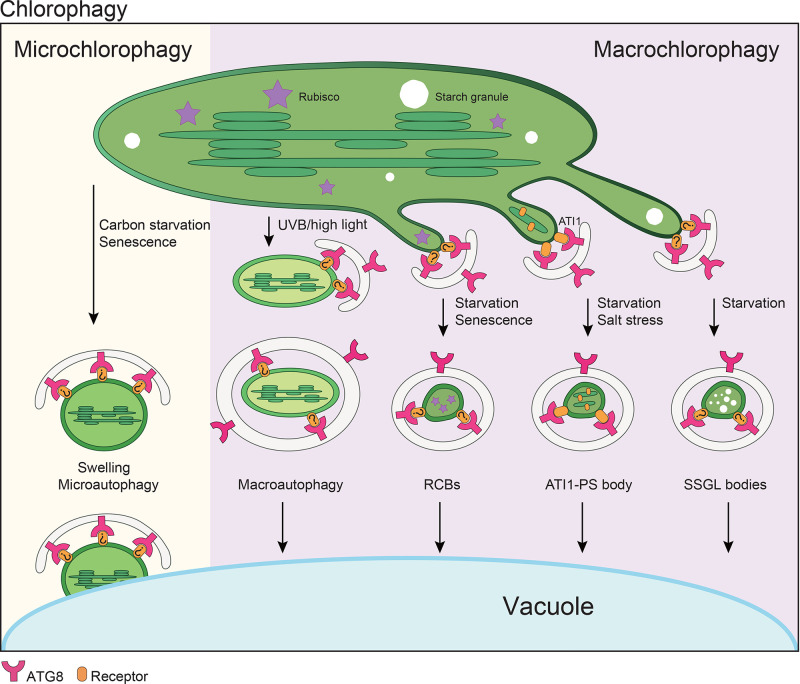
A working model for plant chlorophagy Microchlorophagy mediates whole chloroplast degradation upon carbon starvation and senescence. Macrochlorophagy mediates degradation of whole chloroplasts or chloroplast fragments via several mechanisms, including: Rubisco-containing bodies (RCBs) that are induced by carbon or nitrogen starvation; ATI-PS bodies that are induced by starvation or salt stress; small starch granule-like (SSGL) bodies that are induced during dark-induced senescence or starvation.

Chloroplast components, or even entire chloroplasts, can be degraded by both plastidic and extraplastidic pathways. The extraplastidic degradation of chloroplasts includes autophagy-dependent mechanisms, including entire chloroplast degradation and piecemeal degradation (Figure 5), and autophagy-independent mechanisms, including senescence-associated vacuoles (SAVs) and CHLOROPLAST VESICULATION (CV)-containing vesicles [79]. Using electron microscopy, entire chloroplasts were found in the vacuoles of senescing leaves [80], and accumulation of chloroplast-associated components (stroma, chlorophyll pigments, and Rubisco-containing bodies (RCBs)) was also observed in the vacuoles of WT Arabidopsis cells, but not in *atg* mutants, suggesting that the autophagy machinery is involved in chloroplast degradation [81]. A distinct pathway was seen upon disrupting microtubules via silencing tubulin genes or treating with microtubule-depolymerizing agents; autophagosome formation was suppressed, and plastidic starch degradation was impaired. An autophagy-related pathway for clearing these disorganized chloroplasts was observed, in which selective transport of chloroplasts into the vacuole occurred, independent of ATG6, ATG5 and ATG7 [82]. The details of this mechanism are still unclear.

Upon extensive photodamage, entire chloroplasts can be surrounded by autophagosomal structures in the cytoplasm and transported into the central vacuole, which was directly observed using GFP-ATG8a as a marker to label autophagosomal membranes [78]. This degradation of chloroplasts under UV-B or high light intensities is dependent on core ATG proteins (ATG2, ATG5, ATG7), indicating an essential role of chlorophagy in whole chloroplast clearance. Interestingly, in the presence of ConcA to block vacuolar degradation, the GFP-ATG8a fluorescence was more intense on one side of the autophagosomes, suggesting that additional unknown structures are associated with the sequestration of the entire chloroplast [78]. Entire chloroplasts can also be degraded by microautophagy. In high visible light, autophagy-deficient mutants accumulated abnormal swollen chloroplasts [83]. These swollen chloroplasts were partially encapsulated by GFP-ATG8a-marked membrane and then directly engulfed by the vacuole [83]. Intriguingly, this kind of chlorophagy can be suppressed by applying exogenous mannitol to increase the osmolarity outside the chloroplast, or by improving the integrity of the chloroplast envelope via overexpressing VESICLE INDUCING PROTEIN IN PLASTID1 (VIPP1) [83], a protein essential for envelope and thylakoid membrane maintenance [84–86]. The underlying basis for this regulation warrants further investigation.

### Role of ubiquitination in chlorophagy

How chloroplasts are recognized for degradation is still unclear. Chloroplast membrane integrity is affected by various stresses, during which starch levels and granule structure is also changed, and the structure and shapes of chloroplasts are significantly altered, forming excessive stromules or plastoglobules [78,81–83]. How those ultrastructural changes can be recognized by autophagy for subsequent degradation is in most cases unknown. In yeast cells, selective autophagic degradation of mitochondria involves ubiquitination, but whether a similar mechanism can lead to chlorophagy in plants is not clear [78,83]. Genetic screening identified an E3 ubiquitin ligase, PLANT U-BOX4 (PUB4), as required for ubiquitination of chloroplasts, thus mediating their selective degradation [87]. However, several recent studies have in contrast suggested that chlorophagy does not require PUB4-mediated ubiquitination [88,89], and the relevant component(s) for ubiquitination-mediated chlorophagy is therefore yet to be confirmed.

### Rubisco-containing body (RCB)-mediated chlorophagy

Chloroplasts are large and complex organelles, and in addition to degradation of entire chloroplasts, chlorophagy pathways often function in degradation of parts of chloroplasts via the transfer of bodies containing chloroplast components into the vacuole. RCBs were first identified via immunoelectron microscopy in naturally senescing leaves of wheat (*Triticum aestivum* L.) labeled with antibodies against the large subunit (LSU) of Rubisco. Small spherical bodies containing Rubisco were observed with double membranes [90], and were named RCBs. RCBs contain proteins derived from the chloroplast envelope and stroma, but not from the thylakoid [90]. They usually accumulate in senescent leaves [90–92] or plants under carbon starvation [93] or salt stress [94]. ATG8 co-localized with RCBs upon formation of autophagosomes, indicating that RCBs are delivered to the vacuole by macroautophagy [91]. RCB production is very sensitive to sugar levels [93], and starch content and C/N balance probably affects RCB production *in vivo*. A recent study [95] showed that RCB-mediated chlorophagy is involved in tolerance of Pi starvation, and autophagy-deficient mutants which are unable to form RCBs are extremely sensitive to Pi starvation.

CHARGED MULTIVESICULAR BODY PROTEIN1 (CHMP1A and B), a component of Endosomal Sorting Complex Required for Transport (ESCRT)-III [96], plays an important role in phagophore maturation and efficient delivery of RCBs to the vacuole during chlorophagy. In a *chmp1* mutant, abundant abnormal phagophores, RCB-like bodies and stromal proteins over accumulate [96]. The chloroplasts in *chmp1* contained large starch granules, long extended stromules and interconnecting bridges, which were also found in *atg5* and *atg7* mutants [96]. *chmp1* mutants also over-accumulate peroxisomal and mitochondrial proteins, suggesting that ESCRT mediates autophagic routes for multiple organelles in plants.

### ATI1-plastid associated body (ATI1-PS)-mediated chlorophagy

ATI1 functions in ER-phagy via interaction with the ER, as described above, but also localizes to distinct plastid-associated autophagic structures, termed ATI1-plastid associated bodies (ATI1-PS), of ∼50 to 100 nm diameter [97], containing chloroplast stroma, envelope, and thylakoid membranes. Similar to its role in ER-phagy, ATI1 interacts with ATG8 [38,98] and the core autophagy machinery to mediate partial chloroplast degradation in the vacuole. Under carbon starvation, two distinct bodies, ATI1-ER bodies and ATI1-PS bodies are thus formed, both of which end up in the central vacuole, playing a crucial role in selective turnover of ER and chloroplast proteins, respectively. ATI1-PS bodies also form during heat stress, and plants with reduced *ATI1* expression are hypersensitive to salt stress, indicating a role for ATI1 in salt tolerance [97].

### Small starch granule-like structure (SSGL)-mediated chlorophagy

Finally, an autophagy-related pathway for degradation of plastid starch has been demonstrated. In leaves, plastid transitory starch is the main photosynthetic carbon reservoir, reaching high levels at the end of the day and hydrolyzed into sugars to support plant growth at night [99]. Mutants with abnormal chlorophagy typically also have altered starch levels [93,96,100]. Besides the well-documented plastidic degradation pathway [99], extraplastidic starch degradation can also occur through formation of small starch granule-like structures (SSGLs) in the cytoplasm [100]. SSGLs were found outside of the chloroplast, and localized to CFP-ATG8f-labeled autophagosomes in the cytoplasm and the central vacuole [100]. Moreover, autophagy-deficient mutants have excess starch and a reduction in vacuole-localized SSGLs, indicating that autophagic turnover is an independent and parallel route for degradation of leaf starch [100].

## Future perspectives

It is now becoming clear that plant cell organelles can be selectively degraded by autophagy and autophagy-related processes. These pathways typically require recognition of the organelle, or components of the organelle, to allow selective packaging into autophagosomes for delivery to the vacuole for degradation. Organelle degradation must be tightly regulated to allow disposal of damaged and unneeded organelles, while restraining the pathway from complete organelle degradation, which would lead to cell death. Many unanswered questions remain that will be interesting topics for future research. Why does such a diversity of receptors exist for recognition of some organelles such as the ER? Is this linked to different types of cargo or different stress conditions? Are there as yet unidentified selective autophagy receptors that recognize organelles? Does nucleophagy occur in plants, and if so, what receptor and mechanism is involved? How is the extent of organelle degradation controlled to prevent death of the cell? Answering these questions will provide further insight into the mechanisms of organelle quality control during normal growth and development, and in response to environmental stresses.

## Open Access

Open access for this article was enabled by the participation of Iowa State University in an all-inclusive *Read & Publish* agreement with Portland Press and the Biochemical Society.

## Abbreviations

AIM: ATG8-intearcting motif
ATG: AuTophaGy
ER: endoplasmic reticulum
HL: high light
LSU: large subunit
RCB: Rubisco-containing body
SAV: senescence-associated vacuole
SSGL: small starch granule-like structure
